# Extracts and Gleanings

**Published:** 1873-03

**Authors:** 


### PART II.

### EXTRACTS AND GLEANINGS FROM OUR EXCHANGES.

### *MULTUM IN PARVO.*

A Cure for Corns.—Dr. Barbier, says the *Lyons Medical Journal*, reports the cure of the most refractory corns by the morning and evening application, with a brush, of a drop of a solution of the perchloride of iron. After a fortnight's continued application, without pain, a patient who had suffered martyrdom for nearly forty years from a most painful corn on the inner side of each little toe, was entirely relieved. Pressure was no longer painful, and Dr. B. believed the cure radical. Two other similar cases were equally successful.

In the September number of the same journal, Dr. Topinard, of Paris, adds a long experience confirmatory of the good effects of perchloride of iron in the destruction of corns. He pares off the corns and then applies a drop of the liquid. As it does not adhere readily, it should be repeated and allowed to dry on the part. This requires some patience each time, and the application should be made every two days for a fortnight at least, and in some cases for a month. Even after apparent cure, it should be done from time to time to prevent recurrence of the affection. Sometimes he pares off the corn a second time, taking care not to draw blood, which would render the application painful.

Puerperal Convulsions.—Dr. Emerson (Lynn Medical Society) reported the case, in a primipara, aged thirty. Labor progressed slowly but favorably from ten A.M. of August 11th, to four P.M. of the 12th, when a severe convulsion occurred. She was etherized and delivered with forceps; child healthy. The convulsions continued, and would not yield to chloral; at two P.M. a subcutaneous injection of morphia and atropia was given, controlling the spasms for three hours. Two more injections were given, the last at ten P.M. The patient had no more convulsions and convalesced well.—*Boston Medical and Surgical Journal.*

Aneurism of Arch of Aorta.—Dr. Bartholow met with an aneurism of the arch of aorta in a man about fifty years old. The symptoms were difficult breathing, stridulous cough, swollen face, pupils of unequal size, hoarse voice, occasional distressing paroxysms of dyspnœa and delirium in consequence of carbonic acid poisoning; the left radial pulse was imperceptible, the right was felt distinctly; a tumor presented itself on the upper part of sternum, pulsation well marked under the same, bruit distinctly audible. The dyspnœa was due to the pressure of the tumor on the recurrent laryngeal nerve, the distribution and relations of which are well known. Administered pot. iodid. ℥ ss. *ter die* internally, and ergot in gr. ss. hypodermically, the latter on Langenbeck's plan. The patient recovered rapidly; there remained only some distress of breathing on exertion. He would improve, however, decidedly if he could maintain the recumbent posture.

Dr. Ludlow knew of a case quite analagous to that mentioned by Dr. Bartholow, in which twenty-grain doses of iodide of potassium had been given for 5–6 months with very satisfactory results. The speaker's statement was confirmed by Dr. Graham, the consultant in the case, with a detail of the prominent symptoms.—*Lancet and Observer.*

Treatment of Catarrh of the Lachrymal Sac—Dr. Verneuil, of the Lariboisiere Hospital, believing that lachrymal fistula is due to inflammation of the walls of the sac, and not to the structure of the nasal canal, discards the use of mild injections and catheterism of the lachrymal duct as being almost useless, and employs injections of iodine as follows: Puncturing the tumor, when it exists, by means of the needle of an aspirator, he withdraws its contents and injects the iodine at once, leaving the needle in the puncture, while the syringe is unscrewed to be emptied of pus and filled with the injection. Care needs to be taken not to force the injection through the *puncta lachrymalæ*, since it would produce inflammation of the conjunctiva. When the fistula already exists, and the disease is old, Dr. Verneuil destroys the sac by means of butter of antimony, which is introduced into the sac through a quill after the sac has been properly cleaned. The quill is stopped with cotton wool, and allowed to remain *in situ* for about twelve hours. Subsequently an eschar separates and the wound fills by granulation—*Journal de Méd. et de Chir. Prat.*

Sciatica.—M. Dr. Vergely (Bordeaux) suggests the following method of treatment by external application: Croton oil, fifteen drops, to be spread upon a strip of bandage of cerecloth of the length of the thigh and spread over the course of the nerve.—*Lyon Medicale.*

Asthma.—Dr. Graves (Lynn Medical Society) reported a case of asthma of four years' standing in a child of six years. Nothing had ever given relief, and the attacks had always lasted two or three days. Dr. Graves being called to him in an attack, gave fluid ext. lobelia, gtt. iij., every fifteen minutes, with no relief. Then gave fluid ext. stramonium in the same way, with perfect relief within an hour and a half.

Dr. Webster had found the best results from the use of fifteen to twenty-grain doses of iodide of potassium, with lobelia as a palliative. Has now a case in which he gives a hypodermic injection of morphia whenever an attack comes on, with immediate relief.

Dr. Emerson had treated a case some two years ago with iodide of potass., grs. xx. *ter die*, continued for two or three months, with apparent cure, the disease never having since returned.—*Boston Medical and Surgical Journal.*

Rheumatism.—Dr. Reeves (*Lancet and Observer*) remarks: In the treatment of arthritic rheumatism, the silicate of potash bandage is coming into favorable notice. The parts diseased should be at perfect rest. In order to secure rest, the patient should, if necessary, be splinted down, and not allowed to move about. The chief benefit of opium consisted in the profound rest which it insured. The perfect quiet thus produced permitted the limbs to recover. In many cases of rheumatism we had to contend with the gouty diathesis. In chronic rheumatism of the arthritic variety, with accompanying gouty diathesis, the wine of colchicum acted as a specific remedy. There was nothing like it. There was far less heart complications in these rheumatic cases than was generally supposed. Subacute rheumatism was almost always connected with deranged liver, from malaria. He had found blue mass and quinine quite beneficial in subacute rheumatism.

Treatment of Uterine Hemorrhage by Sulphate of Quinine.—M.M. Gueneau de Mursy and Bartheg have resorted, for several years past, to the use of this remedy in the treatment of metrorrhagia, with marked success. It is principally in hemorrhages which occur several days after delivery, and are accompanied by fever, with daily exacerbations, that they have found its use of most value, and in several crses it has given relief, when used as a *dernier resort*, after ergot and other hemostatics had failed.

In order to obtain the desired results, it is necessary to give the remedy in sufficiently large doses. The gentlemen above mentioned have used it at the rate of eight grains every two hours. Sometimes it is not necessary to continue the remedy beyond a single day, but it is generally prudent to continue it for several days, even though the hemorrhage should have ceased.

Galvano-emesis.—A few days since some children were brought to my house, having eaten poisonous fungi. Two of them were in a state of collapse, almost pulseless, unable to swallow, with the jaws set and frothing at the mouth. None of them had vomited. One of them was apparently dying; and I was preparing to try to assist failing respiration by galvanism, when the thought struck me that, if I could by the same means excite contraction of the stomach, vomiting would probably ensue. I therefore passed one of the poles by a suitable conductor to the top of the œsophagus, and applied the other by a wet sponge to the epigastrium, using the interrupted current. Vomiting was immediately produced in both cases, and a quantity of the fungus was rejected. The children are now well. I am not aware that what I may call "galvano-emesis" has been before made use of, but should think it, from the success which attended my first trial of it, an exceedingly valuable agent in similar cases. I omitted to mention that the children were insensible to the vapor of the strong liquor ammoniæ; it could not, therefore, have been the mechanical irritation which caused the vomiting.—*Fox*, *British Medical Journal.*

Arrest of the Destruction of Lung Tissue in Chronic Phthisis by Means of the Inhalation of Oxygenated Essences.—Dr. Jules Cheron (*Gazette Hebdomadaire*) reports the successful use of the oxygenated essence of camphor in a large number of phthisical cases. He claims to have produced "cicatrizations of cavities of the lungs in chronic phthisis." The following are some of his conclusions: "Preference must be given to the oxygenated essence of *Laurus camphora*, of which the odor is less penetrating than that of *Borneo* camphor, and to the oxygenated essence of cedar, the odor of which is agreeable and sweet and very well supported by the patient." "An intense continued fever, great weakness, rapid progression of the disease, with emaciation, are contradictions for their use." "The slow form of phthisis, with partial conservation of strength, abundant expectoration, with cough and oppression, are the cases in which the best results are obtained." A simple apparatus resembling our ordinary spray producer is used, by means of which the vapor is projected toward the bronchia.

Pruritus of the Meatus.—Dr. Gruber (*All. Wien. Med. Zeitung*) states that the intolerable itching of the external auditory passage, which occurs in paroxysms, often periodical, takes place when uncomplicated by eczema, in persons past the middle period of life, especially those laboring under some disturbance of the circulation. For radical cure, a solution of nitrate of silver, eight grains to the ounce of the distilled water, is to be penciled on the part till the itching no longer occurs.—*Doctor.*

The Treatment of Infantile Paralysis.—Dr. Hitzig and Dr. Jürgensen (*Deutsch. Arch. f. Clin.* 9 B. 3 H.) say that in the period of early childhood there exists a certain disposition to sudden disease of the central nervous system, which in certain cases affects the brain, in others the cord, principally. The difference of the symptoms in palsy takes place chiefly from the different locality affected. The attempt to make sharply-defined definitions of the various kinds of infantile palsy has not succeeded, but quite confused the question. As to what concerns the orgin of the contractions, Hitzig objects to the theory of Hueter, according to which contractions take place according to fixed mechanical rules. External circumstances and mechanical forces may in two cases be precisely similar, and yet in one case a notable contraction will appear which will be absent in the other case. Contractions on the palsied side may, analagously to the cases of degeneration of the cellular tissue of the muscles in experiments when nerves are cut, be explained by the contractions of the scars. As to therapeutics, Hitzig raises an earnest protest against the premature judgment concerning the effect of electro-therapeutics in said disease. In truth the matter so stands, that we can only exceptionally arrive at rapid results; the great majority of cases require, however, a longer treatment of the muscles, in which the signs are found after prolonged investigation, of regained or still present connection with the centre. With great care, in time we arrive at slight results, which are of priceless value to the child. Jürgensen insists that, even when the disease has existed a year, notable advantage is gained by electro-therapeutics. The patience of a horse is required to gain results from electricity. But it is no less true that many cases of infantile palsy which appear hopeless are amenable to galvanism.—*Doctor.*

Calomel in Diseases of the Eye.—A fact not generally mentioned in our works on diseases of the eye, and not very generally known in the profession, I believe, is worthy of notice in reference to the local use of calomel. This remedy is of very great benefit in certain diseases, especially of the cornea, and much used. It should be remembered that it can be placed on the conjunctiva and cornea, not without danger, when the patient is taking the iodide of potash internally. The salts in the tears, and the calomel, often form a caustic sufficiently strong to produce quite a deep and painful eschar.—*Chicago Medical Journal.*

Epilepsy.—Dr. Echeverria (*Medical Times*) says: The formula of the anti-epileptic mixture I have ordinarily prescribed is: ℞.—Potassii bromidi, ʒ iv.— ʒ vj.; liquor. potassæ arsenitis, f ʒ j.; succi conii, f ℥ ij.— ℥ iv.; aquæ dest., ad f ℥ vj. Misce. Sig.—One tablespoonful three times a day, or oftener as required by the case.

EPITHELIOMA CURED BY CREOSOTE—Dr. Forne reports, in the *Montpelier Medical*, a case of epithelioma in the upper lip, of about four centimeters in diameter, cured by the topical application of creosote. The treatment was commenced by dipping a brush in pure creosote, and having removed any that might drop, the whole surface of the ulcer was lightly but firmly touched with the brush, a certain degree of pressure being used, and the brush retained on the same point a few seconds, so as to insure a thorough application. A piece of lint, moistened in a gummy solution of creosote, was then laid upon the ulcer. The application caused but slight pain. Three days subsequently, it was found that the ulcer had not increased. The application was repeated in the same manner, and five times afterwards, at about the same interval. Cicatrization then commenced, and proceeded rapidly, being favored by a dressing of thin paper moistened with a solution of creosote. Entire recovery took place about six weeks after the treatment commenced. *Medical and Surgical Reporter.*

CARBOLIC ACID INTERNALLY.—Dr. Burrall (*New York Medical Record*) says: My own experience would also agree with that of Dr. Bill, that, employed internally, it seems to act "at least as a moderator of pain in cancer."

I would, therefore, venture to suggest it as an internal remedy for the relief of pain. To call it a *peripheral analgesic* would be to give it an odious name, not descriptive of essential effects, although expressive of results which it apparently produces.

When I have used it, it has been almost always in a solution of the sulphate of quinine. It is well to know that a minim of the liquefied crystals weighs about one grain, and from half a minim to a minim three times daily is the commencing dose. This may be gradually increased, although the remedial effects do not seem to correspond with the increase. Not more than one or two drops should be given in the tablespoonful of fluid.

NEURALGIA—GALVANISM.—In order to cure neuralgia by galvanism, we should use the continuous current, by means of small wet sponges attached to small conical conductors. The constant galvanic current has a truly marvelous effect over pain, whereas the interrupted current is of little or no service. The battery used should be Weiss's, sometimes known as Foveaux's. Eight cells of this splendid battery suffice, with very small sponges (about as large as would fill the end of a thimble.) They should be applied to the painful part, an inch or two apart, and moved about, without being actually removed from the skin, for about two minutes. After resting a minute they should then be applied again for two minutes. *Braithwaite.*

Insanity in Children.—Dr. Conyba (*L' Union Med.*, 76, 1872) has seen a great number of cases of insanity in children. Mania is certainly relatively rare in children, but even the few cases of it seen show the erroneous nature of the opinion that mania is quite specially the consequence of unrestrained passion. A disposition to insanity is either hereditary or individual—the latter is shown by the ease with which delirium is aroused by fever. The commonest causes are onanism, pederasty, early menstruation, helminthiasis, dreams, terror, in poison by alcohol or lead, &c., past meningitis, acute exanthems, epilepsy, and imitation in epidemic cases. When children suffer from hallucinations, they have less power against this influence than grown-up persons have; and miraculous cases of religious madness are commonest among children. Folia of children is either general or partial. The general put on the character of depression ot spirit or of mania. In the first case the children sit with sad gaze, with an expression of anxiety in their countenance, turn the head from time to time, as if listening to some one speaking, the pupils are enlarged; they are without fever, but have generally a rapid and bounding pulse. There is sometimes complete intermission, which may conduct to cure. In the maniacal form the children speak much and without any thread to their discourse, concerning persons and things around them; they have illusions and hallucinations, on account of which they whistle, make faces, and laugh or swear, &c. This condition is often quite without fever, but it does not always conduct to lowering of intellect. In partial mania the children have ideas of suicide. Partial mania is often caused by onanism. Such children speak immodestly and make immodest gestures, whilst the genitala bear the marks of onanism. Passive pederasty causes an apathetic, sad, lazy form of insanity, accompanied by bursts of rage and destructive inclination. *Doctor*.

Muriate of Opium.—Dr. Nichol recommends this as the best preparation of opium, never inducing headache. It is made as follows: Take of the best powdered opium, ℥j.; muriatic acid, ℥j.; distilled water, ℥xx. Mix. Shake this mixture very frequently every day, during fourteen days, then strain and filter. The dose is from twenty to forty drops, according to circumstances. Many of my medical friends have tried this preparation, and they highly approve it.

Night Sweats.—Sidney Ringer (*Practitioner*) states that belladonna has a decided effect in checking anomalous cases of habitual sweating; other observers have found atropine in 1-60 grain doses, two or three times a day, to exercise some control over the profuse sweats of advanced phthisis when other remedies had failed.

Coal Oil as a Derivative in Congestion.—D. T. Gilliam, M.D., Nelsonville, Ohio, (*Clinic*, July 20, 1872), has for some time been in the habit of using this article with success, as a counter-irritant and derivative in many internal congestions and inflammations, and especially in diseases of the throat and air-passages, and in inflammation of the lungs. Its action consists in its steady, even, gradual influence over the circulation. It is not uncertain, like poultices, though more efficacious. His method of using it in congestion of the lungs is, to envelop the thorax in woolen cloths saturated with coal oil, and allowing it to remain a sufficient time. In cases of children, he sometimes envelops the entire body in the saturated flannel cloths, but it is more necessary here to watch its effects, as in one case vesication of a large portion of the surface resulted from its too long continuance, though the vesicles were comparatively painless, and healed rapidly upon being sprinkled with flour.

Alcoholic Paraplegia.—Chronic alcoholism affects the whole nervous system, but the spinal cord is the part most prone to suffer. This is not uncommon in females. The condition of the patient is this: She lies in bed or on a couch complaining of severe pains in all the limbs, more especially in the lower ones, which are much wasted, or of a sensation like electric shocks running through them, together with numbness and considerable anæthesia, and at the same time only slight power of movement, or total inability to stand. There is generally enlargement of the liver, with sickness and all the usual signs of chronic alcoholism. The only treatment required is resolutely to break off the stimulants. There need be no fear of inducing delirium tremens, however much has been taken, and however suddenly it is discontinued.—*Braithwaite.*

Cysts in the Liver in Childhood.—Bouchut (*Gaz. des Hop.* 8, 1872) made an observation on a girl of eleven, who was brought into Hôpital des Enfants Malades, with a severe pain in the right hypochondrium, and a swelling in the same locality. The tumor had grown gradually in six months, so that a notable raising up of the ribs was perceived in the region of the liver. The increase in size of the liver was in the right lobe, in which an elastic fluctuating tumor was perceptible to the touch. The child was thin. Dieulafoy's aspirator drew off eighty-five grammes of saltish serosity. A few days after, pleurisy ensued, but the child recovered.

Substitute for Granville's Lotion.—The following is the formula: ℞.—Strongest preparation of aq. ammonia, ℥ iv.; spts. rosemary, ʒ ii.; spts. camphor, ʒ i. Saturate a piece of cotton or lint, and apply immediately with the hand, pressed on it, until slight vesication is produced.

Subcutaneous Injection of Morphia.—Dr. Patterson, of Constantinople, reports that in the late epidemic of cholera at that city, finding all other treatment unsatisfactory, he determined to try the subcutaneous injection of morphia. In the first case a quarter of a grain of the acetate caused relief to the cramps and vomiting in a quarter of an hour, and the skin became gradually warm and moist, and the pulse returned. In ordinary cases he found one or two injections sufficed, in a few three were given, and only once four. He does not maintain that the treatment is a specific against cholera, but that its action is more speedy, certain and effectual than any other tried by him. Out of thirty-two cases in which the treatment had a fair chance, there were only ten deaths. *Braithwaite.*

Inhalation of Bromine.—Dr. Gottwald (*Deutsch Kl.* 1872, 18) has been making experiments with inhalations of bromine, which were recommended by Schutz for croupal and diphtheritic affections of the throat. The inhalations (pure bromine and potassa brom. aa. 0.3, with aq. destil. 150) were employed in eighteen cases of diphtheria and three cases of croup. The diphtheritic cases were all severe cases after measles, typhus, etc. Of these eighteen cases, fourteen recovered and four died, among which two died on the day of admission—one was a convalescent from typhus exhausted by a long-continued abscess of the glands of the neck. In the two cases of croup, the result was admirable and remarkable. Dr. Gottwald, however, did not only use inhalation, but also painting with the solution and cautery with chloride of zinc.

Caffeine.—Dr. H. S. Purdon has been lately trying the hypodermic injection of caffeine in neuralgia from both functional and organic causes. Among his patients at the Belfast General Hospital was a case of "central neuralgia," and which had resisted all plans of treatment. Under the hypodermic use of caffeine, commenced in half-grain doses, increased to one grain dissolved in tepid water, the most satisfactory results were obtained. Caffeine is superior to morphia in never producing any sickness, which is so common when morphia is used hypodermically in any large dose. The alkaloid may also be used in the sleeplessness of delirium tremens.

Fluid Extract of Compound Powder of Jalap and Senna.—I am frequently asked—"What is the best cathartic, moving the bowels speedily, certainly, yet gently?" I answer—a fluid extract of our old compound powder of jalap and senna, made of officinal strength, pound for pound. A teaspoonful is a pleasant and efficient cathartic.—*Eclectic Medical Journal.*

PUERPERAL CONVULSIONS.—Next in order, the attention of the profession was called to the subject of puerperal convulsions and the treatment. No distinction was made in the queries as to the different forms, and the same is true as regards the responses. Eighty-five cases are reported, which gives less than one case in one hundred, and of these, only six cases are reported as fatal. This, to our mind, speaks volumes for the wisdom of the treatment; for no one, not even our enemies, can deny the fact that this is a form of disease in which the tendency is to death and not to spontaneous recovery.

The exhibition of anæsthetics and bromide of potassium in all, and venesection in cases where indicated, seem to be the favorite modes of treatment, and there can be no reasonable doubt of the wisdom of that plan.—*Transactions of the Minnesota State Medical Society,* 1872.

TYPHOID FEVER.—What use is the thermometer in the diagnosis of the typhoid fever? During the first four days the temperature rises one degree each day, starting at 98 3-5 the morning of the first day, but the evening temperature is always two degrees above that of the morning of the same day. The disease is, therefore, not typhoid fever, if, (1) on the second, third or fourth evening the temperature approximates even to the normal (98.6° Fahrenheit); (2) if, during the first two days, the temperature rises to 104° F.; (3) if, before the fourth and sixth days, the evening temperature of a person under middle age does not reach 103°; (4) if the temperature on two of the first three evenings is the same; or (5) if it is the same on the second and the third morning.—*Braithwaite's Retrospect.*

LACTO-PHOSPHATE OF LIME IN TYPHOID FEVER.—Lacto-phosphate of lime is at once an aliment and an article of food, and a medicament of the highest value. It excites the appetite and facilitates digestion. In cases of acute disease, especially typhoid fever and low forms of inflammation, it is most valuable, acting to some extent in the same way as alcohol. The first effect is that the pulse becomes less frequent and the temperature lower. It is especially useful during recovery, being at once the chemical agent of digestion and the natural excitant of nutrition.—*Braithwaite.*

VERATRUM VIRIDE WITH MORPHIA IN PERICARDITIS.—Dr. Lynch (*The Physician and Surgeon,* Baltimore) reports a case of pericarditis treated by veratrum viride, while the patient was under the influence of morphia. The interesting point claimed in this case is, that the entire control of the heart's action may be maintained by the veratrum, notwithstanding its antagonism with the alkaloids of opium.

Hypodermic Use of Ergot.—Reviewing the topic of inertia uteri and the methods adopted to control the hemorrhage, we find the time-honored remedies, cold affusion, styptic injections, manipulation and ergot to prevail, and we beg to call attention to the fact that ergot by the mouth requires, like most other remedies thus administered, from fifteen to thirty minutes to affect the system so as to impress the uterus. In a large share of these cases, the patient would either be safe for recovery by other means, or well nigh beyond the reach of any remedies before this time had elapsed. And here, to our minds, comes in the utility of the more modern method of administering the remedy by the hypodermic method, when we obtain the full effect of the ergot in three minutes, before exhausting hemorrhage and the consequent relaxation is complete, and the life of the patient in extreme danger.—*Transactions of the Minnesota State Medical Society*, 1872.

Vaccination from a Re-vaccinated Person.—Dr. Alexander (Medical Society for Mutual Improvement—*Canada Lancet*) condemned vaccination with virus taken from an adult who had been re-vaccinated, and related an instance of a fatal case of the confluent form of the disease in a medical student, who had not been vaccinated in infancy, but had been vaccinated from an adult who had been re-vaccinated. The Doctor went on to say that the best results had followed in the management of an epidemic at Kingston some years ago from the following treatment: Covering the face and neck with a mask, lined with a composition of carbolic acid, tallow and lampblack, and the stimulating mode of treatment. Dr. Grote advocated the application of undiluted carbolic acid to the pustules individually; it was painless and prevented pitting.

Fluid Extract of Aconite as a Local Application.—Dr. E. J. Marsh (*Eclectic Medical Journal*) says: For two years past I have made numerous experiments with aconite topically applied. After requesting others to use it thus, I am enabled, from their experience and my own, to deduce the following results: All cases of swelled face arising from dental and neuralgic pains, yield readily to a solution of the fluid extract and water, equal parts. It immediately destroys all pain and swelling caused by stings of poisonous insects. Prepared in solution, one part to four of water, it invariably reduces the most obstinate tympanitis in typhoid fever. I order the whole abdomen bathed with it every three or four hours.

Gonorrhœa—Carbolic Acid.—The following is an excellent injection in gonorrhœa: Carbolic acid, eight grains; tannic acid, eight grains; glycerin, half an ounce; water to one ounce.—*Braithwaite's Retrospect.*

Tapeworm.—Turpentine acts with considerable certainty, but is apt to produce intoxication and strangury. Kousso is not easily obtained genuine, and is better, therefore, avoided, as it will be found uncertain in its action. Kameela has an unpleasant griping action, but is otherwise a good remedy. The etherial tincture of male fern is the best remedy. It is efficient and free from the objections to the other remedies. For its successful use the patient should take no food after breakfast except a little mutton broth or tea, and in the evening he should take a brisk aperient. The following morning early, a drachm and a half of the liquid extract or etherial tincture, rubbed up with half an ounce of mucilage, should be taken in about two ounces of milk. It is better to lie quiet for two or three hours after taking this dose, otherwise it is apt to cause nausea and faintness. At the expiration of that time, another aperient dose should, if necessary, be administered, as the male fern itself does not always purge. Milk is the favorite food of the worm, and is, therefore, the best vehicle for the administration of the medicine. After a week's rest, this ordeal should be repeated, in order to be certain that the whole of the worm is expelled.—*Braithwaite.*

Abdominal Aneurism Cured by Aortic Compression.—In the *London Lancet* of April 20, Dr. Walter Moxon reports a case of abdominal aneurism cured by Mr. Durham and himself, by compressing the aorta on the proximal side by means of Lister's abdominal tourniquet, the pad of which was adjusted and screwed down until all femoral pulsation ceased. Compression was steadily maintained for ten hours and a half, the patient being kept under chloroform. No severe constitutional or local symptoms followed. The aneurism after a few hours commenced to pulsate anew, but remained smaller and harder, and gradually grew smaller, so that at the end of a month all pulsation had ceased in it as well as in the femoral.

"Open-Air" Treatment of Hooping-Cough.—In the *Glasgow Medical Journal*, Dr. McLean strongly advocates this plan of treatment, which consists in keeping the little patient as much as possible out-of-doors in the open air. He does not consider this plan of treatment as a specific in every case of hooping-cough, but it is one which, in the hands of a judicious physician, can be made of immense utility, and even in certain complications can be adopted with safety.

Chloride of Zinc and Glycerin in Ophthalmia of New Born Children.—℞. Glycerin, ℥ ss.; chloride zinc, grs. v. Triturate well, and apply a few drops three times a day.—*A. M.*

DEODORIZED TINCTURE OF IODINE.—℞. Tinct. iodini, glycerinæ, aa. f℥ i.; sodæ sulphitis, ʒ i. Rub the salt to a powder in a small mortar, and add the glycerin gradually; then pour in the tincture of iodine, and triturate gently until a solution is effected, and the mixture assumes an amber color.

The above is given on the authority of the *Pharmacist and Chemical Record*, which claims that the properties of the iodine are increased by the addition of sulphite of soda, while the glycerin renders it more convenient for local application. If so, the preparation is certainly an improvement over our present method of bleaching iodine by the addition of aqua ammonia, thus making a solution of iodide of ammonium with an excess of ammonia.—*Northwestern Medical and Surgical Journal.*

TO KILL LICE.—All kinds of lice and their nits may be got rid of, *tuto, cito, et jucunde*, by washing with a simple decoction of stavesacre (*Delphininm staphisagria*), or with a lotion made with the bruised seeds in vinegar, or with the tincture, or by rubbing in a salve made with the seeds and four times their weight of lard very carefully beaten together. The acetic solution and the tincture are the cleanliest and most agreeable preparations, but all are equally efficacious in destroying both the creatures and their eggs, and even in relieving the intolerable itching which their casual presence leaves behind on many sensitive skins. The alkaloid delphinia may be also employed, but possesses no advantage except in the preparation of an ointment, when for any reason that form of application should be preferred.—*Edinburgh Medical Journal.*

THE CAUSE OF SCURVY.—M. Leveir, in a lecture (*Jour. de Med. et Clin. Pract.*) based on an epidemic of scurvy under observation at the hospital at Ivry, maintains that scurvy is due to the absence of vegetables, and vegetables are not indispensable to its removal; but the malady is the result of insufficient alimentation, in bad hygienic conditions. Cold, humidity, excessive work, and depression of spirits, with deficient alimentation, ought to be considered the principal causes of scurvy. In scurvy, the adipose tissue does not disappear; but the muscular system becomes fatty, the muscular striæ being replaced by fatty granulations, even the sarcolemma becoming absorbed. The greatest number of recoveries occur in patients nourished on raw meat, without any intervention of vegetables.

BROWN-SEQUARD'S neuralgic pill is: ℞.—Extract belladon., gr. $\frac{1}{6}$; extract stramon., gr. 1-5; extract cannab. ind., gr. $\frac{1}{4}$; extract aconit., gr. $\frac{1}{3}$; extract opii, gr. $\frac{1}{2}$; extract hyoscyam., gr. $\frac{2}{3}$; extract conii, gr. i.; pulv. glyc., q. s. For one pill.

Chloracetic Acid for Warts.—This acid may be prepared by treating boiling acetic acid (diluted to sp. gr. 1.065) with chlorine in the presence of iodine. A pint of the acid is heated to boiling with forty to sixty grammes of iodine, in a flask retort with long upright neck, and dry chlorine gas passed in through a tube dipping in the liquid. After the chlorine has been passing several days, the liquid is boiled till vapors of iodine appear, then left to cool, and the decanted liquid distilled. It is purified by repeated distillation and crystallization. It crystallizes from liquids distilling between 180° C. and 190° C. It boils at 185° to 187°, and solidifies at 62°.

This acid is recommended as an excellent means of removing warts and other growing excrescences. There are, of course, many ways of preparing it, but the above is believed to be one of the best. If equal weights of hydrate of chloral and fuming nitric acid are exposed in direct sunlight three or four days, till red fumes cease, and then distilled with a thermometer, that going over at 185° C. is nearly pure tri-chloracetic acid.—*Boston Journal of Chemistry.*

Disinfection of Rooms.—Dr. E. Derby (*Boston Medical and Surgical Journal*) says: To completely disinfect a room, including its carpets, furniture and wall-paper, close the doors, windows and chimney; put from one to two pounds of brimstone (according to the size of the room) in an iron pot, pour over it a little alcohol, set it on fire, and leave the room for four hours. This process is injurious to the colors of many fabrics, and to gilded articles, and this injury corresponds in degree with the amount of moisture present in the room.

When disinfection by sulphur fumes is impracticable, the next best thing to do is to wet the carpet, furniture and walls with a strong solution of carbolic acid, one part in fifty of pure acid.

Clothing which can be washed may be disinfected by boiling in water for one hour. Bedding, and clothing which cannot be washed, may be disinfected by exposure for four hours to dry heat at a temperature of 225° to 300° Fahr.

Ginger Wine.—This is an excellent stomachic, and is very popular in England as a cheap substitute for grape wine: Take sugar, twelve pounds; water, three and a-half gallons; ginger, four ounces. Boil them together for half an hour; when cooled to seventy-five degrees, add the rinds of six lemons and some good yeast; let it ferment for ten or fourteen days, then add a pint of brandy and bottle it for use.

Itch Ointment.—℞. Sublimated sulphur, ℥j.; subcarbonate of potash, ℥ss.; adeps simplex, ℥iv. Apply morning and evening.

Warm Bath in Insanity and in Burns.—Dr. Wilkins, in his report on Insanity, to the California Legislature, refers to the warm bath as a favorite treatment in Italy, and in some parts of Holland and France. He often saw a dozen patients in one bath room, with their heads alone in sight, the bathing tub being covered, except a hole for the head. There they usually remain for from one to three hours, in some instances from six to eight hours, and occasionally for days at a time. Dr. Gudden, of Zurich, kept a man thus immersed for five days, on account of excitement connected with bed-sores. The patient is reported to have slept well during a portion of the time, and to have been cured of the sores. No exhaustion or ill consequence followed. A case is related of a man scalded by steam, and not insane, who was placed by Hebra in a tepid bath, and kept there for three weeks, until a new cuticle had formed over the entire surface. This patient recovered without inconvenience. The water was kept at a temperature most agreeable to the patient. Thus employed, it is said to relieve effectually the extreme pain from the burns.—*Boston Journal of Chemistry.*

Gelseminum in Urethro-Cystitis.—Dr. A. K. Webster (*Journal Materia Medica*) says: I desire to call the attention of the profession to the use of gelseminum in urethro-cystitis. Within three months I have treated nine cases with this drug; each case was characterized by incontinence of urine, frequent, scanty and painful micturition, with symptoms of local urethritis—also great prostration of strength.

No external applications were used, and no medicine except the following prescription: ℞. Fld. ext. gelseminum (Tilden's), ʒ ij.; bromide potassium, ʒ iij.; aquæ menthæ pip., ℥ iiss. M. Sig. Take teaspoonful every three hours.

To their great surprise and gratification, they were relieved in twelve, and were able to resume labor in forty-eight hours. I had used bromide of potassium in similar cases before, with little or no avail.

Elixir of Pepsin and Bismuth.—Emil Scheffer has shown, by a series of experiments, reported in the *American Journal of Pharmacy* for August, that pepsin is precipitated from its solutions by the salts of bismuth, and that the elixirs of pepsin and bismuth which are offered by several manufacturers must owe any efficiency they possess to the alcohol and bismuth they contain.

Bisulphide of Carbon.—Dr. Cowling (*American Practitioner*) calls attention to this rather neglected but really efficacious local anæsthetic. Our own experience is confirmatory of Dr. Cowling's, as it is of value in relieving pain of a neuralgic character.

Carbolic Acid in Skin Diseases.—In an interesting work "On the Treatment of Skin Diseases," by Dr. McCall Anderson, (published by Macmillan & Co., and for sale by Williams & Co., Boston), which contains no less than *eleven thousand* consecutive cases, we find the following with regard to the use of carbolic acid in these affections:

"*Carbolic acid* has a less disagreeable odor than the tarry preparations, and is more cleanly. It is soluble in water with the aid of a little glycerin or spirit, and forms a colorless watery solution. For these reasons it can often be used when tar is inadmissible, as when an eruption is situated upon an exposed or hairy part. . . The following is a form in which I frequently prescribe it: Crystallized carbolic acid, two drachms; pure glycerin, six drachms; rectified spirit, four ounces; distilled water, one ounce; dissolve. Sponge the affected parts two or three times daily, and also when itching is complained of. This solution is of use in cases of chronic erythema, chronic eczema, and the like, and not only relieves irritation of the skin, but also is directly curative."—*Boston Journal of Chemistry.*

For Chapped Hands and Lips.—The following recipe for chapped hands was much approved during the siege of Paris: Tincture of aloes, two to four parts; glycerin, thirty parts. M. On retiring to bed, a piece of cloth wet with this is to be applied over the chapped places, and the hands then gloved.

A good recipe for chapped lips is the following: Spermaceti, four drachms; white wax, one drachm; oil of almonds, two troy ounces; glycerin, one troy ounce. Melt the spermaceti, wax and oil together, and when cooling stir in the glycerin and perfume.

Creosote in Anthrax.—Dr. F. R. Millard, Beetown, Grant county, Wisconsin, uses creosote in the treatment of anthrax. His plan is to incise to about three-fourths of the depth of the induration, and introduce a pledget of lint saturated with creosote. He renews lint twice daily for a few days, and removes with the scissors all dead tissue. In the course of four days he begins to use pressure by adhesive plaster. As constitutional measures he advises quinine with acid sulph. aromat., or tinct. ferri chlo. and wine.

Phosphorus Pills.—A writer in the *Druggist's Circular* gives the following formula for a pill of phosphorus, by which he says they can be made of small size, at short notice, and to keep without evolving fumes: Dissolve one grain phosphorus in half a drachm of chloroform, and rub in a mortar with two scruples powdered liquorice root till all the chloroform has evaporated. Add half a drachm powdered soap and work into a mass with water, and divide into twenty-four pills.

Bodily Quietude in the Diseases of Children.—Dr. Henry Blanchard, in the *Boston Medical and Surgical Journal*, says: "I desire to bear testimony to the importance and value of one other principle in the management of the diseases under consideration. It is to be regarded of paramount importance: namely, quiet of body—*rest*. Highly important in most diseases, it is indispensable in some. I do not fail to enjoin it most strictly in all cases affecting the abdomen. Even in cases not grave in character, a quiet, recumbent position greatly facilitates recovery. I dislike to have a dysinteric patient rise from the bed during the course of the disease. Children in their summer complaints are equally benefited by a recumbent posture and entire quiet. I know it is not practicable to carry out this management with children always, but much may be done by perseverance and patience in this direction. Tossing babies about in the manner of some nurses and mothers, with the idea of easing their suffering and quieting their cries, must be looked on as an evil and useless habit. Cradles and other rocking machines should be condemned as worse than useless. The quieter children are kept, the less suffering and the less demand for medicine."—*Northwestern Medical and Surgical Journal.*

Alcohol and Renal Disease.—It seems probable, from discussions which have taken place lately in the *London Lancet* and other British journals, that the profession has been in error in regarding the immoderate use of alcoholic drink as tending to produce kidney disease. Dr. Dickinson has been at considerable pains to collect statistical information on this point; he has also looked into the whole subject very closely, and has arrived at the conclusion that drinking habits are not, on the whole, great contributors to the general mortality from kidney disease. This view is diametrically opposed to a wide-spread medical belief, and one which is sure to meet with considerable opposition. It is a well-known physiological fact that only a small quantity of the alcohol taken into the system is eliminated by the kidneys. It cannot be, therefore, that the cells of the uriniferous tubes are over-taxed in the elimination of alcohol. Besides, it has never been proved, either by clinical observation or by statistical evidence, that there is any decided tendency of alcohol excess to produce kidney disease. The subject is a very important one, and requires careful investigation. *Canada Lancet.*

Ulcerative Stomatitis.—Dr. Fordyce Barker says: In the treatment of this affection, for a few years past, I have mainly relied upon the sulphites either of magnesia or of soda, and I have cured my cases more rapidly than I formerly did, when I depended upon the chlorate of potassa, borate of soda, etc.

Constipation Relieved by Electricity.—Dr. Webber reported (Suffolk District Medical Society) the case of a woman, sixty years of age, who, for thirty years, had constipation of the most aggravated character. She had taken enormous amounts of medicine, but had never obtained relief to the bowels without enemata and the persistent use of cathartics. On the first of October, Dr. Webber had commenced the use of the Faradaic current, applying one pole in the lumbar region, and the other on the abdominal walls. It was repeated on the next day, and on the following day the patient had one or two discharges. The treatment was continued for two or three weeks, until she had had fourteen applications, since which time the discharges had been natural. She is now considered cured. It was noticed at first that the patient was tolerant of an exceedingly strong current, but that as the bowels began to regain their contractility, the anæsthesia disappeared. Dr. Webber had seen six cases of a similar character lasting a longer or shorter time and yielding to electricity. In one case diarrhœa had been excited, which ceased on stopping the electricity. All these cases showed a greater or less tolerance to a powerful current as the constipation increased or diminished.—*Boston Medical and Surgical Journal.*

Mixed Vapors for Anæsthesia.—Dr. J. D. Davis (*Medical Record*), in commenting upon this subject, remarks that the mixture recommended by the Committee on Anæsthetics of the American Medical Association deserves to be more universally known and used, its advantages being—the small quantity required; the rapidity of its action; the shortness of the stage of excitement (it often being wanting entirely); the absence of distressing after-effects; its comparative safety over chloroform; being compounded in definite proportions, it can be scientifically administered; the pungent, stifling odor of the ether and the burning sensation of the chloroform are to a great extent overcome; the sedative effects of chloroform are modified by the exciting influence of ether, while to both is added the stimulant alcohol. The mixture is composed of alcohol, one part; chloroform, two parts; and ether, three parts. It is easily remembered by taking the initials in the order of the alphabet: A., C., E., the corresponding number of parts being 1, 2, 3.—*Medical Examiner.*

Erethema or Simple Inflammation of the Skin.—One grain of sulph. atropia rubbed up with an ounce of glycerin forms an excellent application to inflammation of this character.

Eczema.—The citrine ointment diluted with olive oil, one to two or four, to which two grains of morphia to the ounce is added, often affords a cure or prompt relief in this perplexing disease.

Measles and Pneumonia.—Dr. N. S. Davis (*Chicago Medical Examiner*) remarks: When symptoms of pneumonia occur in connection with measles, the best remedy in children is a combination like the following: ℞. Liq. ammon. acetas, ℥ iss.; syrup ipecac, ℥ ss.; tinct. opii et camph., ℥ i.; tinct. verat. viride, ʒ i. The dose proportioned to the age of the child. For a child two years old, about twenty drops are usually necessary, though it is best to begin with ten drops. For an adult, one teaspoonful. It should be given every two, three or four hours till the fever is controlled. In the active stage of the disease, while using this mixture, cover the chest externally with fomentations.

I have considerable faith in the popular notion about onions. They certainly afford more relief to the breathing than any other thing we can use. I attribute it to the impregnation of the air which is inhaled with the volatile oil, more than to any absorption from the surface of the chest, and think this application preferable to blistering.

It will be advisable, in these cases, to give a powder, containing from half a grain to a grain of calomel, with Dover's powder, according to the age and restlessness of the patient, about three times a day. The liquid mixture may be continued, at longer intervals, until the symptoms of pneumonia have entirely disappeared.

Treatment of Constitutional Syphilis in Infants.—Administer to the mother and child a bath of corrosive sublimate of the following proportions, viz.: corrosive sublimate, from a half to one ounce; alcohol, four ounces, to an ordinary bath. If the child is nursed by its mother, give her daily a pill of one grain of protoiodide of mercury; but when, on the contrary, the child is not suckled, order the child every day two and a half drachms of syrup of sugar, and twenty drops of the following solution: Corrosive sublimate, twenty grains; water, two pounds eight ounces. Mix. Each dose will thus contain one-fiftieth of a grain of corrosive sublimate.

Happy Combination for Chronic Diarrhœa.—Rayer recommends the association of cinchona, charcoal and bismuth in the treatment of chronic diarrhœa, in the following proportions: ℞. Subnitrate of bismuth, ʒj.; cinchona, yellow, powdered, ʒ ss.; charcoal, vegetable, ʒj. M. chart xx. S. Two or three a day during the intervals between meals. Nitrogenous diet with raw meat if necessary.—*Union Medicale.*

Glycerin Lemonade in Diabetes Mellitus.—O. Schultzen recommends the following, which is to be taken during the day: Glycerin fifty grammes, water one thousand, citric or tartaric acid five grammes.

CHLORAL AND ITS ADMINISTRATION.—The disagreeable sensation produced in the throat by chloral, and the difficulty experienced in getting some patients to swallow the substance in its usually nauseous form, have induced Mr. S. Limousin to experiment on a new method of administering it, *i. e.*, in the form of capsules with a gelatinous or saccharine envelope. His process is published in the *Journal de Pharmacie et de Chimie.* Although it is easier to introduce the chloral into capsules of soft gelatine, yet it is necessary to use the hard, because the former induce too rapid decomposition. Medical testimony shows that chloral administered in this manner has its full effect, and can be swallowed without any sense of irritation in the throat. Moreover, if the chloral employed in making these capsules is in any respect impure, if it has not been re-distilled, if it contains free hydrochloric acid or an excess of moisture, the gelatine is rapidly attached and the capsule destroyed, incontestible evidence being thus offered of the condition of the chloral.

OPIUM IN WOUNDS OF THE INTESTINES.—At the Northeastern Indiana Medical Society (*Detroit Review*) two cases were reported: 1. A girl, aged two years, fell on the sharp corner of a cellar door, rupturing the walls of the abdomen and the colon, so that the bowel protruded. Opium and perfect rest secured recovery. 2. A woman, attempting suicide, opened the abdomen near the umbilicus with a knife, and cut away several inches of the colon. The ends were returned and the wound dressed, and a full dose of opium given, the woman being then left to die. But she recovered.

POISONOUS EFFECTS OF ZINC UTENSILS.—The *Union Medical* calls attention to a new source of danger, caused by the substitution of zinc for tin in the manufacture of pots and pans by traveling tinmen. Zinc sheet can be had at seventy centimes the kilogramme, while tin costs three or four francs, so that it is often substituted in the making of kitchen utensils. The fraud cannot be detected by the eye, but a little vinegar boiled in the vessel will immediately corrode the surface, and, if done in the process of cookery, will give rise to symptoms of poison.

FOR HOOPING-COUGH.—A writer recommends the following formula for hooping-cough: ℞. Potassæ bicarb., gr. xij.; cocc. cacti, gr. x.; potass. bromid., gr. xvj.; t. belladonnæ, gtt. xx.; t. cardamomi comp., ℥ i.; aq. cinnam., q. s., ℥ ij. For a child a year or two old, the dose is one teaspoonful every three or four hours. We are told that, although this formula must not be regarded as a specific, it is very effective and superior to anything in ordinary use.—*Medical and Surgical Reporter.*

REDUCTION OF PARAPHYMOSIS.—The reduction of paraphymosis is always found to occasion very severe pain. On being called to a case the other day, in a boy of about six years old, it occurred to me that I might much facilitate the operation and reduce the pain by letting some of the serum escape from the much swollen cellular tissue. I accordingly punctured the prepuce in eight or ten places with a fine needle. A good deal of serum immediately escaped; the remainder came away with great facility as soon as I commenced to make pressure preparatory to the reduction. The result surpassed my anticipations; I reduced the paraphymosis with the greatest ease, and without the slightest wincing or expression of pain on the child's part.

Perhaps this "wrinkle" regarding the alleviation of suffering in a very painful manipulation may be worth recording in your columns.—*Ashe, Medical Press and Circular.*

SENNA-COFFEE.—It may not be generally known that the disagreeable taste of infusion of senna may be completely removed by the addition of coffee in its preparation.

For a full dose, take a teacupful (say one ounce) of senna leaves, a heaped teaspoonful (say two drachms) of freshly-parched and ground coffee, and boiling water a sufficient quantity to make a teacupful (say four fluid ounces) of infusion—steep till of sufficient strength.

To the infusion thus prepared, add milk and sugar to taste. The drink will be quite acceptable to adults, and not disagreeable to children.

INGROWING TOE-NAIL.—Dr. Stillwell, of Epsom, (*British Medical Journal*) says that, for the removal of this affection, his "invariable mode of proceeding has been to find the edge of the nail with a probe, and then to remove the granulations and hypertrophied cellular tissue, on both sides, if requisite. In no case have I been disappointed, or ever had to treat the patient for the return of this grievous complaint.

CIMICIFUGA RACEMOSA IN SMALL POX.—Dr. Norris, (*Transactions Alabama State Medical Association*) extols this plant as a preventive of small pox, and also as a curative. He adds that he could not succeed in vaccinating persons who were drinking daily of tea made of the root. But it is so easy to be misled as to the effects of agents on the human body.

A SATURATED tincture of the fresh root of stillingia, in doses of from five to fifteen drops, three times daily, is said to be a valuable remedy in the various forms of tertiary syphilis. Its efficiency in the many forms of skin diseases is undoubted.

